# Tunisian *Toxoplasma gondii *strains genotyping by the use of AK69 marker

**DOI:** 10.1186/1756-3305-4-167

**Published:** 2011-08-26

**Authors:** Sonia Boughattas, Rym Ben-Abdallah, Emna Siala, Imen Ben-Abda, Olfa Souissi, Karim Aoun, Aida Bouratbine

**Affiliations:** 1 Laboratoire de Recherche 05SP03, Laboratoire de Parasitologie, Institut Pasteur de Tunis, 13 Place Pasteur BP74, Tunis Belvédères 1002, Tunisia

**Keywords:** AK69, high sensitivity, mixed genotype, *Toxoplasma*

## Abstract

**Background:**

Clinical manifestation due to infection by *Toxoplasma gondii *is closely linked to the infecting strain of the parasite. Several genetic markers are available to determinate its genotype but few of them are able to discriminate between the three predominant lineages, namely types I, II and III. The number of markers decreases when atypical, recombinant/mixed genotypes need to be identified.

**Findings:**

In our study, the contribution of sequence polymorphisms in the AK69 gene as typing markers for *T. gondii *was investigated for the first time in an epidemiological study. The coding region of the marker was amplified, sequenced and aligned for different *Toxoplasma *strains. The identified nucleotide polymorphism at 12 positions was able to highly discriminate between the different congenital toxoplasmosis Tunisian strains. Moreover the high detection sensitivity level of the marker enabled unambiguous identification of mixed/recombinant genotypes directly.

**Conclusion:**

It can be, thus, very useful for direct typing in areas where such genotypes are frequently encountered, mainly in the African continent.

## Findings

*Toxoplasma gondii *(*T. gondii*) is an intracellular protozoan that infects all warm-blooded animals including humans. It is considered as one of the most widespread parasites in the world [1]. While three genetic lineages I, II and III are predominant in Europe and North America [2], recombinant strains, are rather more frequently observed in North Africa [3].

Different methods are used for genotypic characterization of *T.gondii *[4,5]. A collection of more than 200 markers, allowing direct genotyping, was described [6]. Nevertheless most of them are biallelic and could distinguish just two of the 3 clonal types at a single locus. Only a small percentage of markers presents two biallelic polymorphisms that lie closely by each other and are able to identify even recombinant strains. AK69, one of these markers, located on chromosome X, is the only one supposed to distinguish between the different types using a single restriction enzyme [6].

The aim of this study is to investigate the AK69 marker performance and contribution to the direct characterization of recombinant strains in Tunisia.

This study included 3 reference strains: RH (Type I), PRU (Type II) and NED (Type III) provided by CRB Toxoplasma (France) and 14 clinical specimens (11 Amniotic Fluids, 2 Placentas and 1 new born Cerebral Spinal Fluid) isolated from confirmed congenital toxoplasmosis cases [3]. These isolates were characterized previously by multilocus analysis at six polymorphic markers [3].

DDesigned primers were used in a PCR assay to amplify the polymorphic region of AK69 gene. AK69Fex TTGAACATCTGGTGCGAGAC and AK69Rex GTCTCCCAACCACCTCCATA were used as external primers and AK69F ACGAGCAACCATATCTTACC and AK69R CGAACGGACAACAAGCTA as internal ones. The annealing temperature was of 55°C for the first round amplification and 58°C for the second amplification round. A starting 1:100 dilution of primary PCR products was necessary for the second amplification round. Reaction was carried out in a final volume of 25 μl as described elsewhere [3].

PCR products were purified by GenElute PCR CleanUp Kit (Sigma Aldrich), and directly sequenced by Applied Biosystems 3130 Genetic Analyzer. Both forward and reverse PCR internal primers were used for the sequence reactions. SNPs have been identified, once sequences aligned.

The AK69 amplified products were also systematically digested with HinfI as recommended by the supplier (Invitrogen). The restriction fragments polymorphism within the locus was resolved by 3% agarose gel, stained with ethidium bromide and visualised under UV.

To test the sensitivity of the marker, control RH strain DNA was quantified by spectrometric method at the wave length of 260 nm and then diluted into aliquots from 100 ng to 1 fg using 1:10 dilutions. One micro-liter of each concentration was used in the PCR assay.

The amplified DNA by AK69 external primers gave a product of 633 base pairs. Small DNA amounts or no amplification products were detected after the first amplification round. The external primers were used simultaneity with other primers in our multiplex assay [3].

In the second round of amplification, positive reaction was obtained from all positive tested samples producing a 490 bp band. Negative controls (Water, Extracted no DNA, Negative sample) remained free of amplified products.

To test the sensitivity of the PCR assay, different quantities of *T. gondii *DNA were used. Analysis of the serial dilutions of *T. gondii *RH pure genomic DNA established a detection limit of the PCR assay at 100 fg.

Sequences analysis of the three reference strains [GenBank : HM003613, HM003614 and HM003615] showed several closely spaced polymorphisms by the presence of 12 substitutions nucleotide (at the positions 111, 125, 142, 155, 195, 215, 221, 250, 291, 324, 326 and 378) resulting in three-way typing. It reveals four, one and two HinfI restriction sites for type I, II and III respectively.

The differential RFLP patterns visualized on the gel confirmed sequencing results and allowed easy and unambiguous discrimination of the three strains.

**Figure 1:**
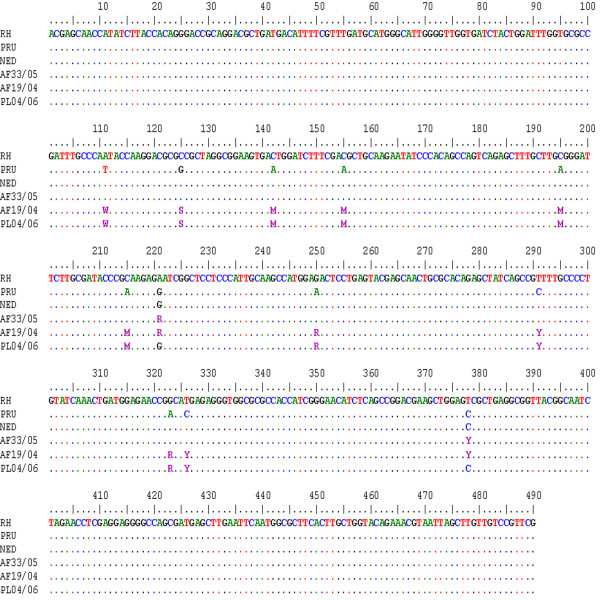
**Multiple alignment analysis at AK69 PCR product sequences**. Sequences are from the 3 archetypal types I, II, and III (RH, PRU, and NED respectively) and mixed clinical Tunisian specimens I/III, I/II and II/III (AF33/05, AF 19/04 and PL04/06 respectively). Periods (.) indicates identical nucleotides related to the sequence of Type I (first row).

The restriction patterns analysis of AK69 for 13 clinical samples was unequivocal: two samples had type I allele (AF06/06, AF44/05), two others had type II allele (AF10/08, AF16/07) and three samples type III allele (CSF01/08, AF08/08, AF07/06). Five of the remaining strains revealed mixed profiles between two types: three strains (AF26/04, AF08/06, and AF33/05) were typed I/III, two strains (PL05/06, AF19/04) typed I/II and one strain (PL04/06) was typed II/III. These results have been confirmed by direct sequencing of the PCR products: the SNPs of the corresponding two types were superposed on the same sequence [GenBank: JN82707, JN82708, JN82709 respectively] (Figure 1). The fourteenth isolate AF07/02 shows after restriction unique profile (u-1) including the common band of 199 bp and unusual one of approximately 270 bp. The corresponding sequence [GenBank: HM003616] revealed just two restriction HinfI sites at 199 and 221 positions suggesting thus the concomitant presence of type I and II. These results are in concordance with previous multilocus typing of the clinical samples (Table 1).

**Table 1:** Summary of AK69 genotyping results and previous multilocus typing of Tunisian clinical strains

	Alleles							
	
Samples	AK69	3'SAG2	5'SAG2	SAG3	GRA6	BTUB	APICO	FINAL
AF06/06	1	1	1	1	1	1	1	I
AF44/05	1	1	1	3	1	1	1	I/III
CSF01/08	3	1	1	3	1	1	1	I/III
AF08/08	3	1	1	3	1	1	1	I/III
AF26/04	1+3	1	1	3	1	3	1	I/III
AF08/06	1+3	1	1	3	1	3	3	I/III
AF33/05	1+3	1	1	1+3	1	3	1	I/III
AF07/06	3	1	1	3	1+3	1	1	I/III
PL05/06	1+2	1	1	2	1+2	ND	1	I/II
AF07/02	1+2	1	1	ND	1+2	1+2	1	I/II
AF19/04	1+2	1	1	1+2	1+2	1+2	1	I/II
PL04/06	2+3	1	1	2+3	1	ND	1	I/II/III
AF10/08	2	1	1	2+3	1	ND	1	I/II/III
AF16/07	2	1	1	1+2+3	1	ND	1	I/II/III

We report the first use of AK69 marker in an epidemiological study. Very little is known about this marker: its chromosomic location and abbreviation which was suggesting its putative function: adenosine kinase, despite the fact it was described for the first time in 2005 during the genome map realization of the *Toxoplasma gondii *[6]. Isolates previously studied were typed to individual level without ambiguity by this three-way marker. The results were in perfect agreement with the previous multilocus DNA analysis with regard to the power of identifying and differentiating these isolates. Indeed, the use of this marker in typing Tunisian strains revealed an interesting approach as it allowed solely, direct identification of final genotype in the half of the cases which is higher than other marker's rate (Table 1). In the other cases, it detected just one of the corresponding allele. Its ability to be used in a multiplex PCR reaction [3] is a significant advantage since the rate of identification of the final genotype exceeds 70% with the supplementary use of GRA6 marker and reaches 100% with the use of SAG3 marker concerning Tunisian isolates. Our strains were typed to individual level without ambiguity by these three markers summarizing thus the result obtained by a 7 locus analysis.

Our results showed that the AK69 marker offers a new tool for direct identification of strains with promising alternative for recombinant ones even the small sample size which is explained by the low rate of the parasite detection during pregnancy in our country (these samples were retained positive from 243 analyzed specimens). Despite this, the performance of the AK69 marker could be very helpful especially in geographic areas with frequent mixed/recombinant strains like Africa continent [3]. Its use in a multiplex PCR reaction presents a further considerable advantage.

In addition, the detection limit of AK69 marker was shown to be 100 fg of pure toxoplasmic DNA, which corresponds to one *T. gondii *organism [7]. AK69 gene marker was thus revealed to be the most sensitive described RFLP marker since it is more sensitive that SAG3 marker: detection limit 1 versus 5 to 10 parasite [4], making it more suitable to clinical samples with low parasite load.

Moreover the Blast X of our sequences demonstrated a perfect match with putative ROP8 protein. This rhoptry antigen belongs to ROP2 family [8]. This later is known as a major virulence component and is involved during the invasion process of host cells as well as crucial biological functions [9].

## Conclusion

The molecular approaches presented in our work offer an easy and a highly sensitive discrimination of the different *T. gondii *strains even in presence of mixed or new types. A potential implication of this marker in virulence pathway is also suggested.

## Competing interests

No conflict interest is declared.

## Authors' contributions

SB carried out the molecular studies, the sequence alignment and drafted the manuscript. RBA and ES have made substantial contributions to acquisition of data and their analysis. IBA have been involved in drafting the manuscript and revised the English typing. OS carried out qPCR for diagnosis. KA has been involved in revising the manuscript critically for important intellectual content. AB conceived the study and has given financial support. All authors approved the final version of the manuscript.
